# Cryo-electron microscopy structures of human thyroid peroxidase (TPO) in complex with TPO antibodies

**DOI:** 10.1530/JME-22-0149

**Published:** 2023-01-24

**Authors:** Stuart Baker, Ricardo Núñez Miguel, Daniel Thomas, Michael Powell, Jadwiga Furmaniak, Bernard Rees Smith

**Affiliations:** 1 FIRS Laboratories, RSR Ltd, Parc Ty Glas, Llanishen, Cardiff, UK

**Keywords:** thyroid peroxidase, autoimmunity, autoantibodies, cryo-electron microscopy, structure

## Abstract

Determination of the structure of the extracellular domain of human thyroid peroxidase (hTPO) by cryo-electron microscopy (cryo-EM) is described. TPO, purified to homogeneity was complexed with the hTPO monoclonal autoantibody 2G4 Fab and also with a mouse monoclonal TPO antibody 4F5 Fab (which competes with autoantibody binding to TPO). Both complexes were analysed by cryo-EM. The two structures (global resolution 3.92 and 3.4 Å for the 2G4 complex and 4F5 complex, respectively) show TPO as a monomer with four domains; the N-terminal domain, the peroxidase domain (POD), the complement control protein (CCP)-like domain and the epidermal growth factor-like domain which are all visible in the structures. The relative positions of the domains are fixed with a disulphide bond between cysteine residues Cys146 in the POD and Cys756 in the CCP domain preventing significant flexibility of the molecule. The entrance to the enzyme active site, the haem group and the calcium binding site are clearly visible on the opposite side of the TPO molecule from the 2G4 and 4F5 binding sites. Extensive interactions are seen between TPO and the two antibodies which both bind to distinct epitopes on the POD domain, including some residues in the immunodominant region B mainly via different residues. However, the epitopes of the two antibodies contain three shared TPO residues. This is the first high-resolution structure of TPO to be reported and it should help guide the development of new inhibitors of TPO enzyme activity for therapeutic applications.

## Introduction

Thyroid peroxidase (TPO) is a key enzyme in the production of thyroid hormones (Taurog *et al.* 1990) and a major thyroid autoantigen (Czarnocka *et al.* 1985) in addition to thyroglobulin (Roitt *et al.* 1956) and the thyroid-stimulating hormone receptor (Rees Smith & Hall 1974).

The TPO molecule is a glycosylated, transmembrane protein expressed predominantly on the apical surface of thyroid follicular cells facing the thyroid lumen. It is a multi-domain protein consisting of an N-terminal domain, peroxidase domain (POD), complement control protein (CCP)-like domain, epidermal growth factor (EGF)-like domain and the transmembrane domain with a cytoplasmic tail (Ruf & Carayon 2006).

To date, the structure of TPO has not been solved despite numerous attempts (Gardas *et al.* 1997, Hendry *et al.* 1999, Hendry 2001*a*, Thomas 2019, Williams *et al.* 2020), and in order to study the relationship between TPO structure and function, several models of TPO have been produced (Hendry 2001*a*, Hobby *et al.* 2000, Le *et al.* 2015, Williams *et al.* 2018, 2020).

We now report the cryo-electron microscopy (cryo-EM) structures of human TPO (hTPO) bound to TPO antibodies (TPOAb); the human monoclonal TPOAb (hMAB) 2G4 and the mouse monoclonal antibody (mMAB) 4F5.

## Materials and methods

### TPO preparations

hTPO extra-cellular fragment (amino acids 1–839) was produced in High Five (*Trichoplusiani*) insect cells (Life Technologies) as described previously (Grennan Jones *et al.* 1996) and purified by ion exchange chromatography, immuno-affinity chromatography and size exclusion chromatography (SEC). The purified recombinant human TPO (rhTPO) in 50 mmol/L NaCl, 20 mmol/L Tris-HCl, 0.1 mmol/L KI pH 7.4 (TPO buffer) was stored at −80°^o^ in aliquots of 2 mg/mL (concentration determined using an extinction coefficient of 1.333 at 280 nm).

Haem incorporation was assessed as the ratio of OD at 412 nm to OD at 280 nm and purity was determined by sodium dodecyl sulphate-polyacrylamide gel electrophoresis (SDS-PAGE) (Laemmli 1970).

Peroxidase activity was assessed using a guaiacol oxidation assay in which samples (100 µL) of different concentrations of TPO were diluted in TPO buffer with 0.25 mg/mL bovine serum albumin (BSA), mixed with 2 mL of guaiacol solution, (43 mmol/L guaiacol, 10 mmol/L Tris–HCl pH 8.0, 0.25 mg/mL BSA) and incubated at 37°C for 20 min. After the addition of hydrogen peroxide (10 µL, 60 mmol/L), the absorbance at 470 nm was recorded at 15, 30 and 60 s. The guaiacol activity (guaiacol units/mL (GU/mL)) was calculated from the individual change in absorbance at OD 470 nm with 1 GU equivalent to a change in OD 470 nm of 1.00 per minute. The specific enzymatic activity was expressed as GU/mg by dividing GU/mL by sample TPO concentration.

TPOAb binding activity was tested in an inhibition ELISA using reagents from RSR Ltd (Cardiff, UK). In the assay, dilutions of purified TPO (from 800 to 3.1 ng/mL in 150 mmol/L sodium chloride, 20 mmol/L Tris–HCl pH 7.8, 5 mg/mL BSA, 9.6 mmol/L sodium azide, 0.1% (v/v) Tween 20) were incubated with pooled TPOAb-positive patient sera or controls and incubated for 1 h at room temperature. The pre-incubated TPO/TPOAb mixture was added to rhTPO-coated ELISA plate wells and incubated for 1 h at room temperature with shaking. After washing, goat anti-human IgG-horseradish peroxidase conjugate (Life Technologies) was added to each well and incubated with shaking at room temperature for 30 min. After further washing, the reaction was developed using 3,3',5,5'-tetramethylbenzidine (TMB substrate solution, K Blue Advanced, Neogen, Ayr, UK) followed by stop solution (0.25 M H_2_SO_4_) and the OD 450 nm and OD 405 nm were measured on a plate reader. The TPOAb binding activity in a sample was determined from a calibration curve prepared from a master lot of purified rhTPO.

### TPO hMAB 2G4 and mMAB 4F5 preparations

2G4 Fab was produced from 2G4 IgG (RSR Ltd) using papain from Papaya latex (Sigma-Aldrich) using an IgG:papain ratio of 50:1 w/w (Horimoto *et al.* 1992). Digestion reactions were incubated at 37°C in PBS (8.1 mmol/L Na_2_HPO_4_, 1.5 mmol/L KH_2_PO_4_, 2.7 mmol/L KCl, 137 mmol/L NaCl pH 7.4) plus l-cysteine (8.25 mmol/L) and ethylenediaminetetraacetic acid (1.65 mmol/L) for 4 h with occasional mixing by inversion. The reactions were stopped by the addition of iodoacetamide (to 47 mmol/L) and incubation at room temperature for 30 min.

4F5 Fab was produced from 4F5 IgG (RSR Ltd) by papain digestion with 30:1 w/w IgG:papain ratio as described for 2G4 IgG earlier (Hendry 2001*a*, Hendry *et al.* 2001*b*).

Digested 2G4 IgG or 4F5 IgG mixtures were purified on a MabSelect column (Cytiva, Little Chalfont, UK) in 150 mmol/L sodium chloride, 1 mol/L glycine, pH 8.6. The non-binding fractions were collected as the Fab pool and the concentrations were determined from the absorbance at 280 nm using an extinction coefficient of 1.333 mol mg^−1^ cm^−1^ as determined by ProtParam.

The 2G4 Fab and 4F5 Fab were analysed by SDS-PAGE (Laemmli 1970) and by analytical SEC.

### TPO-2G4 and TPO-4F5 complex preparation

TPO and 2G4 Fab were mixed to give a molar ratio (TPO:2G4 Fab) of 1:1.9 and incubated for 1 h at room temperature. The complex was then separated from the unbound components using SEC. The fractions representing the complex were pooled and concentrated using Vivaspin 6 centrifugal concentrators (Sartorius, Epsom, UK) to 2 mg/mL and used for cryo-EM studies. The concentration of the complex was determined by spectrophotometry at 280 nm using an extinction coefficient of 1.623 mL mg^−1^ cm−^1^ as determined by ProtParam. Enzymatic activity of the complex was determined by guaiacol oxidation assay as described earlier and analysis by SDS-PAGE and analytical SEC was also performed.

TPO-4F5 complex was prepared using the same procedure, concentrated to 2 mg/mL and analysed as described above for TPO-2G4.

### Cryo-EM grid preparation and data collection

For the preparation of cryo-TEM grids, 100 mmol/L 3-((3-Cholamidopropyl)dimethylammonio)-2-hydroxy-1-propanesulfonate (CHAPSO) was added to the TPO-2G4 complex or to the TPO-4F5 complex to a final concentration of 8 mmol/L, and then 3 μL of the mixture was applied to UltrAufoil and Quantifoil R1.2/1.3 300 mesh grids. A total of six cryo-TEM grids were prepared using Vitrobot Mark IV (Life Sciences). The data were collected from the UltrAufoil grid from undiluted samples (2 mg/mL).

The data for both complexes were collected on a Titan Krios 300kV with Falcon3 detector in counting mode (Cryo-EM Facility, Department of Biochemistry, University of Cambridge, UK) using 96,000× magnification, corresponding to a pixel size of 0.827 Å/pixel. A total dose of 45.4 e/Å^2^ (TPO-4F5 complex) and 43.8 e/Å^2^ (TPO-2G4 complex) and 40 fractions were used for the recording of movies during each exposure which lasted 50 s (TPO-4F5 complex) and 47 s (TPO-2G4) and a defocus ranging from −2.4 to −0.9 μm with a defocus step of 0.3 μm. Automated data acquisition was performed using EPU software (Thermo Fisher Scientific).

A total of 1949 movies for TPO-2G4 and 1726 movies for TPO-4F5 were collected during 48 h. Pre-processing and particle picking were performed using Warp (Tegunov & Cramer 2019) identified about 191,000 particles for TPO-2G4 and 313,000 particles for TPO-4F5 which were extracted in boxes of 340 pixels and imported to Cryo-SPARC processing for 3D reconstruction (Punjani *et al.* 2017). Briefly, the picked particles were subjected to 2D classification. Classes representing TPO complexes were used to generate ab-initio models which were then used as starting models for heterogenous refinement. The best particles and volumes from heterogeneous refinement were finally refined using non-uniform refinement.

### Model fitting and refinement

The experimental maps for both complexes show TPO bound to 2G4 or to 4F5. For the TPO-4F5 complex, the models of antibody (PDB-Id: 1AIF) and TPO (AlphaFold database, https://alphafold.ebi.ac.uk/, Tunyasuvunakool *et al.* 2021) were fitted into the cryo-EM density. In the case of the TPO-2G4 complex, the cryo-EM structure of TPO from the TPO-4F5 complex and the antibody (PDB-Id: 1AIF) were fitted into the cryo-EM density.

The structures were manually refined using COOT v0.9 (Emsley *et al.* 2010). Then, the Refinement Cascade protocol within the Discovery Studio 2021 suite of programs (BIOVIA Dassault Systemes 2021) was run to refine automatically the structures of both complexes. Finally, six rounds of minimization were run with MODELLER (Sali & Blundell 1993, Webb & Sali 2016) for both complexes.

## Results

### TPO-2G4 and TPO-4F5 complexes

**Figure 1:**
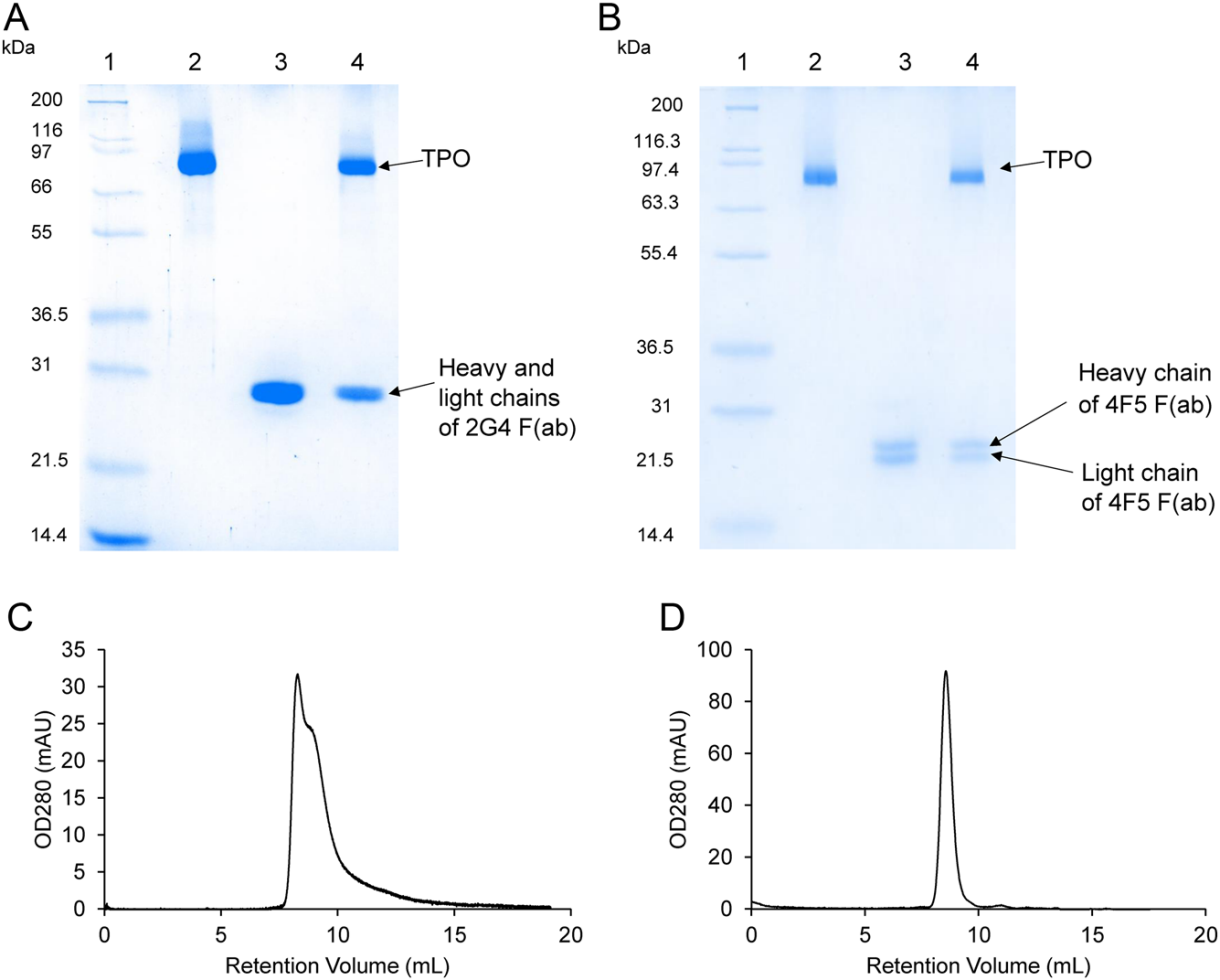
(A) Analysis of purified TPO-2G4 complex by SDS-PAGE (12% acrylamide gel) under reducing conditions. The positions of the molecular weight standards are shown and the positions of the TPO and 2G4 Fab heavy and light chain fragments are marked (lane 1: molecular weight standards; lane 2: purified TPO; lane 3: purified 2G4 Fab; lane 4: TPO-2G4 complex). (B) Analysis of purified TPO-4F5 complex by SDS-PAGE (12% acrylamide gel) under reducing conditions. The positions of the molecular weight standards are shown and the positions of the TPO and 4F5 Fab heavy and light chain fragments are marked (lane 1: molecular weight standards; lane 2: purified TPO; lane 3: purified 4F5 Fab; lane 4: TPO-4F5 complex). (C) Analysis of purified TPO-2G4 complex by SEC (TSKgel 3000SW column run in 50 mmol/L NaCl, 20 mmol/L Tris-HCl, 0.1 mmol/L KI pH7.4). The absorbance at OD 280 on the vertical axis is measured in milli-absorbance units (mAU) using an analytical AKTA system (1 mAU is 1/1000th of an absorbance unit i.e. OD 0.001) for the complexes in (C) and (D). (D) Analysis of purified TPO-4F5 complex by SEC (TSKgel 3000SW column run in 50 mmol/L NaCl, 20 mmol/L Tris-HCl, 0.1 mmol/L KI pH7.4).

The purified TPO-2G4 Fab complex at 2.0 mg/mL concentration had an OD 412 nm/OD 280 nm ratio of 0.318 and a specific activity of 993 GU/mg. The purified TPO-4F5 Fab complex at 2.0 mg/mL concentration had an OD 412 nm/OD 280 nm ratio of 0.288 and a specific activity of 1060 GU/mg. On reduced SDS-PAGE, the complexes were separated into two clear components corresponding to rhTPO at a molecular weight of approximately 97 kDa and bands representing the heavy chain (HC) and light chain (LC) of the Fabs at approximately 25 kDa (Fig. 1A and B). The purified preparations of both complexes resolved as >95% heterodimeric complex on analytical SEC (Fig. 1C and D).

### Human TPO structure

**Figure 2:**
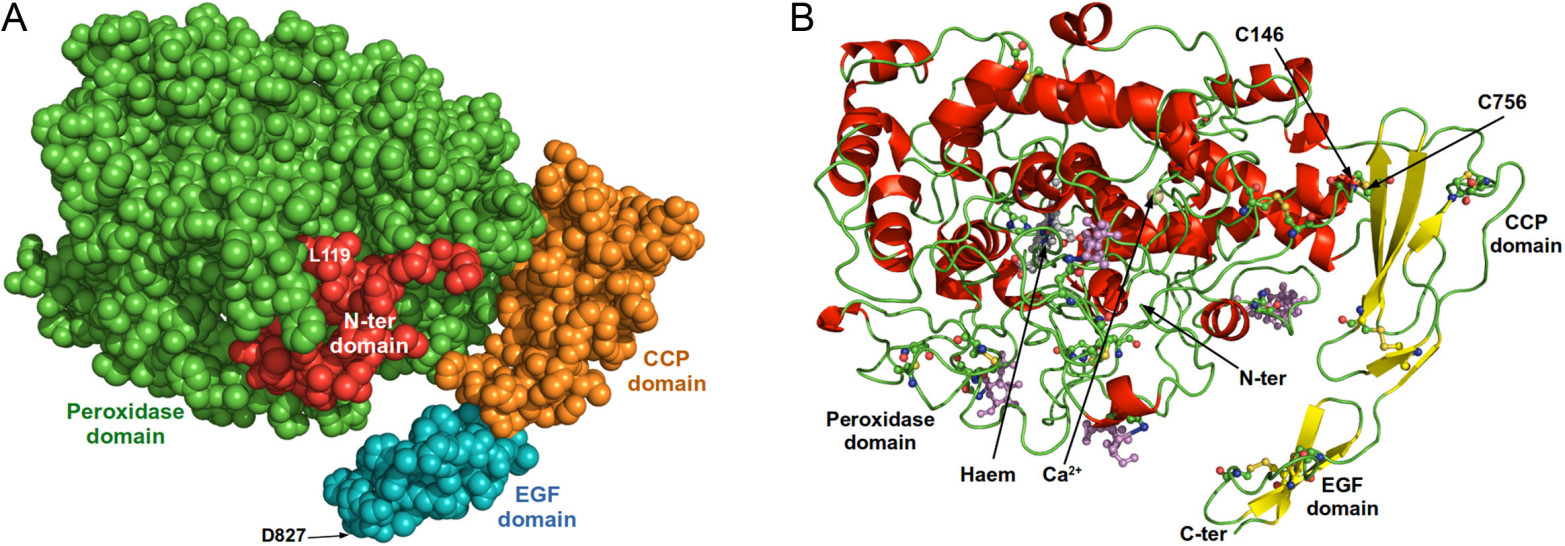
Structural features of TPO. (A) TPO structure (amino acids Leu119 to Asp827). The structure is in a space-fill representation with the N-terminal domain in red, the peroxidase domain (POD) in green, the complement control protein (CCP)-like domain in orange and the epidermal growth factor (EGF) domain in blue. (B) TPO structure (amino acids Leu119 to Asp827). The structure is in a cartoon representation with the disulphide bonded cysteine residues and glycosylation sites in ball and stick representation with oxygen in red, nitrogen in blue and sulphur in yellow. The glycans are shown in ball and stick in purple. The POD, CCP and EGF domains and the disulphide bond between Cys146 in the POD and Cys756 in the CCP domain are marked. The N- and C-terminals are marked.

**Figure 3:**
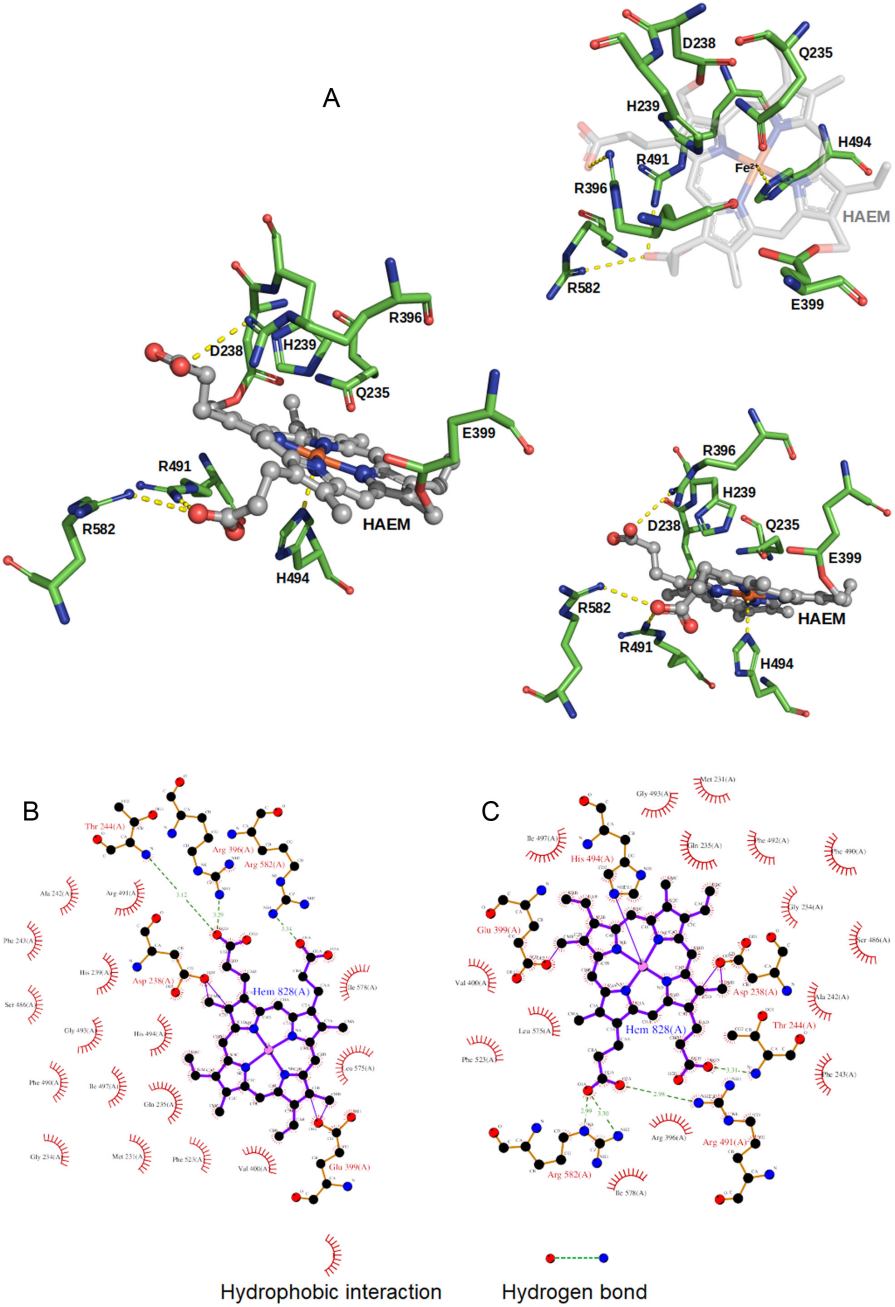
A. The TPO active site showing, the haem group in a ball and stick representation with carbon atoms shown in grey, hydrogen atoms in blue and the iron ion in orange. Residues Asp238 and Glu399 which are covalently linked to the haem group and Arg396, Arg491 and Arg582 that bind to the carboxylate groups of the haem group are shown, as well as the proximal histidine, His494, that binds the iron ion in stick representation. Residues lining the active site, located at the distal side of the haem group, are shown, including the distal histidine, His239. B. Schematic Diagram of the residues from TPO and the haem group interacting in the TPO-2G4 complex. C. Schematic Diagram of the residues from TPO and the haem group interacting in the TPO-4F5 complex.

The structure of hTPO bound to 2G4 Fab was solved by cryo-EM at a global resolution of 3.92 Å and the structure of TPO in complex with 4F5 Fab to a global resolution of 3.4 Å (Supplementary Table 1 and Supplementary Fig. 1, see section on supplementary materials given at the end of this article). The solved structures show the TPO molecule from Leu119 to Asp827 for both complexes. Our TPO structure is composed of four domains; the N-terminal domain is visible from residues 119 to 141 with residues 109–118 not visible in the structure. The whole POD amino acids 142–734, a 6 amino acid linker between the POD and CCP domains and the CCP domain amino acids 741–795 are visible while the EGF domain is visible from amino acids 796–827 with amino acids 828–839 not visible in the structure (Fig. 2A and B, Supplementary Fig. 2). The relative positions of the POD and CCP domains are fixed with a disulphide bond between cysteine residues Cys146 in the POD and Cys756 in the CCP domain. The haem and the enzyme active site (Fig. 3) are both located on the POD (composed of 593 residues). The POD secondary structure consists predominantly of α-helices, some of which surround the haem group (Fig. 2A and B, Supplementary Fig. 2). The CCP domain consists of several β-strands running parallel to the long axis of the domain with several conserved hydrophobic residues at its centre (Supplementary Fig. 2). The EGF domain is made of a two-stranded β-sheet. There are two intra-domain disulphide bonds joining four cysteine residues in the CCP domain (Cys742 to Cys782 and Cys768 to Cys794) and two disulphide bonds (Cys800 to Cys814 and Cys808 to Cys823) in the EGF domain (Supplementary Fig. 2).

Cryo-EM density is observed at all four N-linked glycosylation sites Asn129, Asn307, Asn342 and Asn569, all in the POD of TPO. Although this cryo-EM density is visible in both TPO complex structures it is better defined in the TPO-4F5 structure and shows 1, 2, 5 and 4 sugar molecules attached to the asparagine residues at positions 129, 307, 342 and 569, respectively.

### Structure of TPO enzyme active site

The haem group in the TPO enzyme active site is bound to the TPO molecule by two covalent bonds with Asp238 and Glu399. Furthermore, the two carboxylate groups of the haem form salt bridges with Arg396, Arg491 and Arg582 of TPO. In addition, there are interactions of the proximal histidine (His494) with the iron ion (Table 1 and Fig. 3). The TPO active site is located at the distal side of the haem group with Gln235, Asp238 and Glu399 lining the active site together with the distal histidine His239 and Arg396. Other TPO residues that contact the haem group are shown in Table 1.

**Table 1:** TPO residues which interact with the haem group.

Main type of interaction	In the TPO-2G4 complex	In the TPO-4F5 complex	Main type of interaction
Induced dipole	Met231	Met231	Induced dipole
Hydrophobic	Gly234	Gly234	Hydrophobic
Hydrogen bond	Gln235	Gln235	Hydrogen bond
Covalent bond	Asp238	Asp238	Covalent bond
Polar	His239	-	-
Induced dipole	Ala242	Ala242	Induced dipole
Induced dipole	Phe243	Phe243	Hydrogen bond
Hydrogen bond	Thr244	Thr244	Hydrogen bond
Salt bridge	Arg396	Arg396	Salt bridge
Covalent bond	Glu399	Glu399	Covalent bond
Hydrophobic	Val400	Val400	Hydrophobic
Hydrophobic	Leu403	-	-
Induced dipole	Ser486	Ser486	Polar
Induced dipole	Phe490	Phe490	Induced dipole
Salt bridge	Arg491	Arg491	Salt bridge
-	-	Phe492	Hydrophobic
Hydrophobic	Gly493	Gly493	Hydrophobic
Fe coordination	His494	His494	Fe coordination
Hydrophobic	Ile497	Ile497	Hydrophobic
Hydrophobic	Phe523	Phe523	Hydrophobic
Induced dipole	Leu575	Leu575	Hydrophobic
Induced dipole	Ile578	Ile578	Induced dipole
Salt bridge	Arg582	Arg582	Salt bridge

His494 is the proximal histidine that binds the iron ion.

### TPO calcium binding site

The TPO calcium binding site has pentagonal bipyramidal geometry and is located in a loop between POD residues 321 and 327 with residues Asp240, Thr321, Phe323, Asp325 and Ser327 binding the calcium ion (Supplementary Fig. 3).

### Interaction of 2G4 Fab and 4F5 Fab with TPO in the complexes

The Fab preparations of 2G4 and 4F5 are visible in the structures bound to TPO and both are typical Fab fragment structures. The 2G4 Fab consists of HC residues Glu1 to Ser215 and LC residues Asp1 to Gly212 while 4F5 Fab consists of HC residues Asp1 to Asp207 and LC residues Ala(-1) to Cys214.

Extensive interactions between 2G4 Fab and TPO and 4F5 Fab and TPO are clearly visible in the cryo-EM structures of the respective complexes (Table 2). The interface surface area for the TPO-2G4 complex (2148 Å^2^) is larger than for the TPO-4F5 complex (1959 Å^2^) (Supplementary Table 2). Contributions to the interface surface area are greater from the HCs than the LCs in the case of both antibodies, although the difference is more marked for the TPO-4F5 complex (Supplementary Table 2). The number of interactions between 2G4 and TPO (*n* = 148) is greater than between 4F5 and TPO (*n* = 120). Furthermore, 2G4 produces 13 strong interactions including salt bridges, ion pairs and hydrogen bonds compared to 8 strong interactions in the case of 4F5. In contrast, 4F5 is involved in more polar interactions (*n* = 23) with TPO than 2G4 (*n* = 13) (Tables 2 and 3). The antigen binding site of 4F5 is rich in aromatic residues (*n* = 14). However, no aromatic interactions are present between 4F5 and TPO. There are four aromatic interactions between TPO and 2G4 which has a total of nine aromatic residues in its CDRs (Table 2 and 3).

**Table 2:** TPO residues that contact antibody heavy (H) and light (L) chains. Residues with atoms separated by less than 4 Å are considered to be contact residues.

TPO - 2G4 complex		Common TPO residues	TPO - 4F5 complex	
2G4 chain	TPO residues		TPO residues	4F5 chain
-	-	-	L177	L
H	G194	-	-	-
H	L196	-	-	-
L	N198	-	-	-
H	G199	-	-	-
L,H	F200	-	-	-
H	P201	-	-	-
H	E207	-	-	-
H	E276	-	-	-
H	E277	-	-	-
-	-	-	E461	H
-	-	-	Q465	H
-	-	-	R595	H
-	-	-	E596	L
-	-	-	F597	L,H
-	-	-	C598	H
-	-	-	G599	L,H
-	-	-	P601	L,H
-	-	-	R602	L
-	-	-	L603	H
L	E604	E604	E604	L,H
L	T605	-	-	
L,H	P606	-	-	
L	A607	A607	A607	H
L	D608	D608	D608	H
-	-	-	S610	H
-	-	-	T611	H
-	-	-	A612	H
L	R616	-	-	-
L,H	D620	-	-	-
L,H	L623	-	-	-
H	D624	-	-	-
H	L625	-	-	-
L,H	K627	-	-	-
L	H628	-	-	-
-	-	-	P645	H
-	-	-	K659	L
**Total residues**	**21**	**3**	**19**	**Total residues**

**Table 3:** Number and type of interactions made in the complexes between TPO and the two antibodies.

Type of interaction	2G4	4F5
Salt bridge	3	2
Ion pair	3	1
Hydrogen bond	7	5
Polar	13	23
Hydrophobic	39	28
Aromatic	4	-
Induced dipole	70	57
Cation-π	2	1
Long-range charge	7	3
**Total**	**148**	**120**

**Figure 4:**
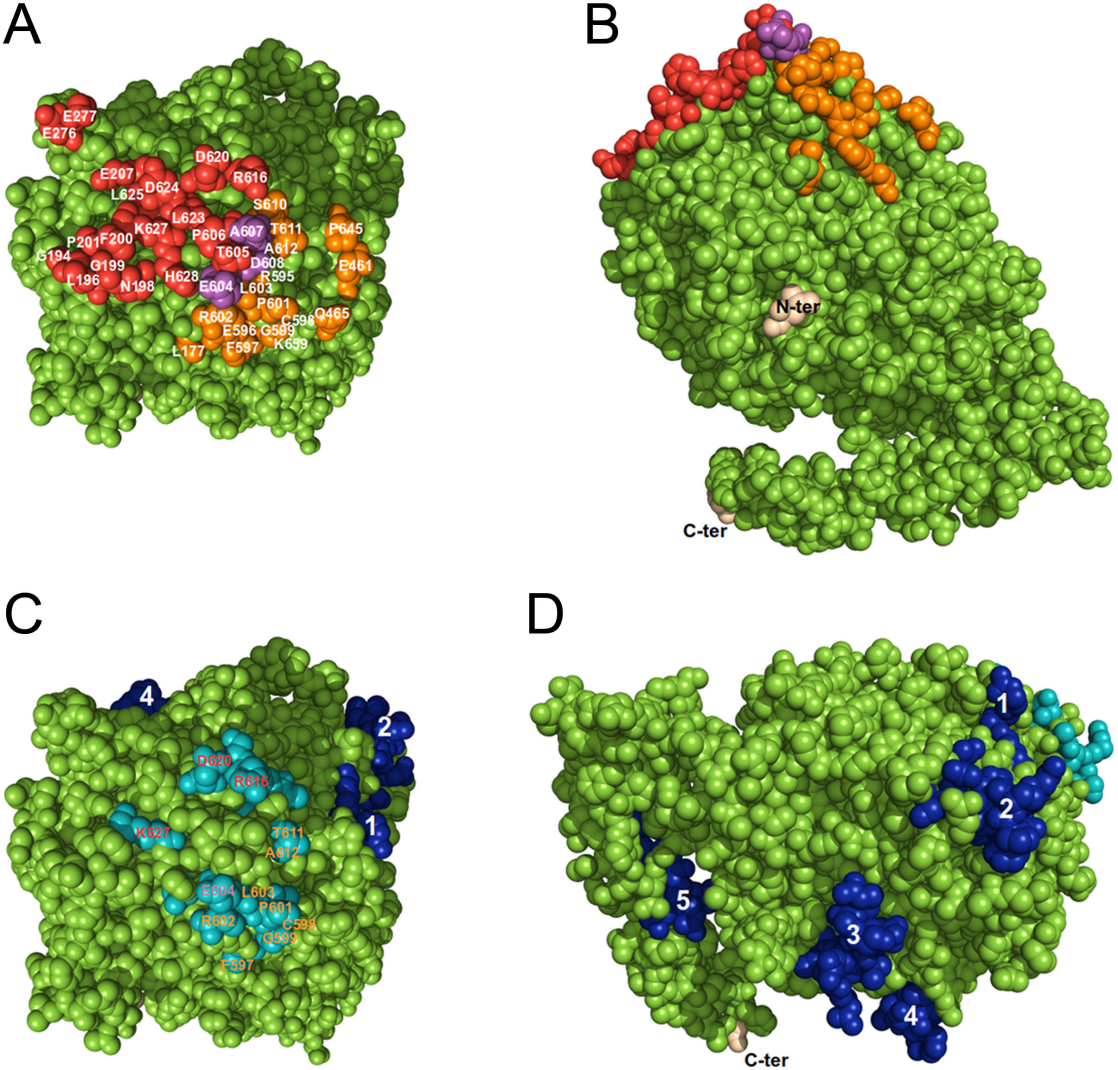
(A) and (B) 2G4 and 4F5 epitopes on the TPO structure in two views rotated by approximately 90° along the horizontal axis. The structure is in space-fill representation with TPO residues that interact with 2G4 shown in red and TPO residues that interact with 4F5 shown in orange. TPO residues that interact with both antibodies are shown in violet. The N- and C-termini are marked in (B). (C) and (D) The immunodominant regions A (IDR-A, in dark blue) and B (IDR-B, in light blue) are shown on the TPO structure in two views. IDR-A is composed of five separated clusters of residues, labelled 1–5. IDR-B residues that interact with antibody 2G4 are marked in red and IDR-B residues that interact with antibody 4F5 are marked in orange in (C). IDR-B Glu604 which interacts with both antibodies is marked in purple.

**Figure 5:**
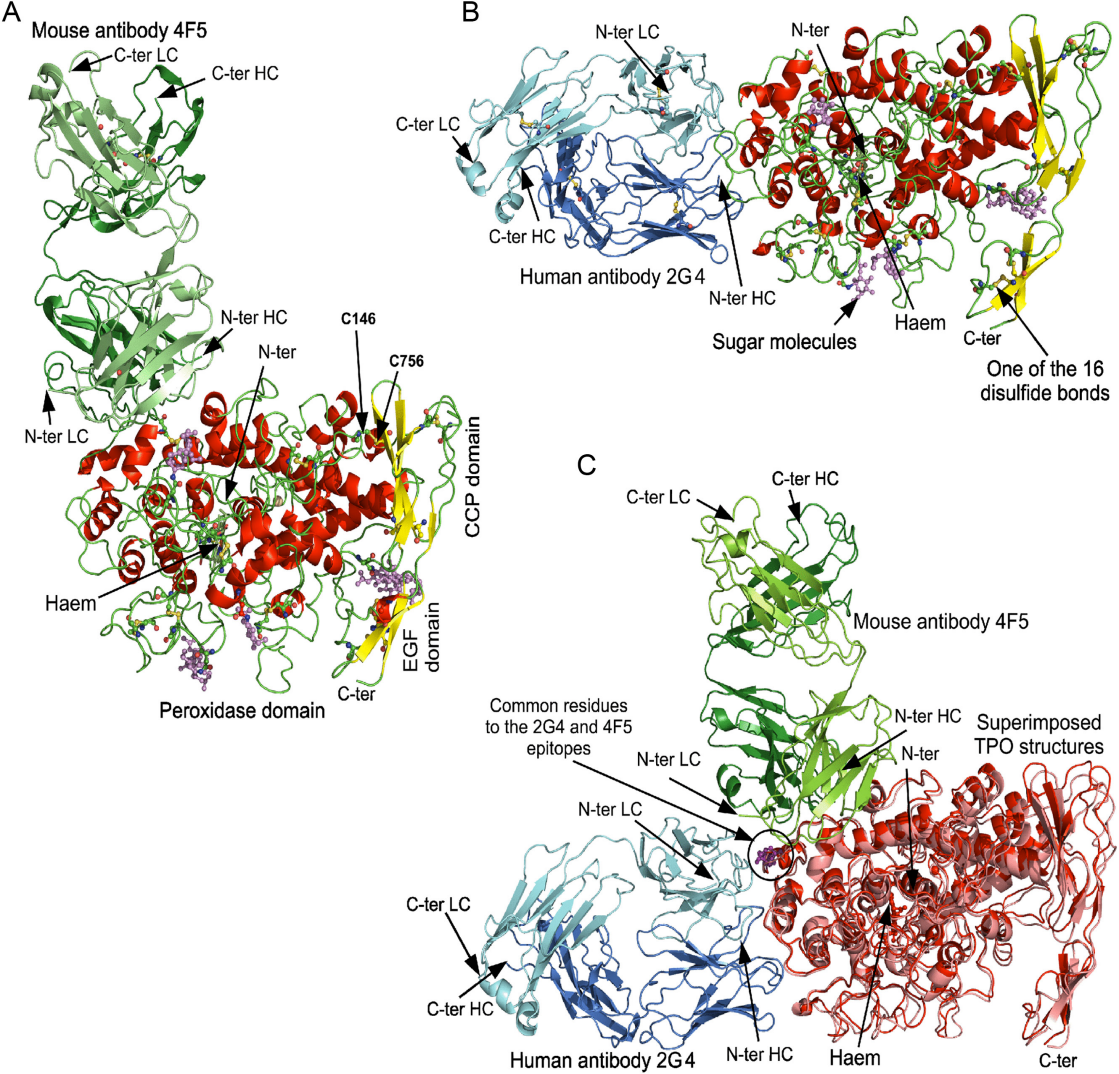
Cryo-electron microscopy structures of the extracellular domain of thyroid peroxidase in complex with mMAB 4F5 Fab and hMAB 2G4 Fab. (A) TPO-4F5 complex. (B) TPO-2G4 complex. (C) Superimposition of the coordinates of TPO from the two complexes showing different orientations of the two antibodies with respect to TPO. The three TPO residues which interact with both 4F5 and 2G4 are shown in ball and stick in purple. Molecules are shown as cartoon representations. Cysteines involved in disulphide bonds, asparagine residues bonded to glycans and sugar molecules are shown as ball and stick. Oxygen atoms are shown in orange, sulphur atoms in yellow and nitrogen atoms in blue. Carbon atoms are shown in green when located in a loop, red when located in a helix, yellow when located in a strand and violet when located in a sugar molecule. The glycans are shown in ball and stick in violet.

The discontinuous, conformational 2G4 Fab epitope on TPO consists of three regions (amino acids: 196–207, 276–277 and 604–628) with 21 TPO residues forming interactions with the antibody (Fig. 4A). The discontinuous, conformational epitope of 4F5 Fab on TPO also consists of different regions (amino acids: 177, 461–465, 595–612 and 645–659) forming interactions with 19 residues on TPO (Table 2, Fig. 4B). There are three common residues, Glu604, A607 and Asp608 on TPO that are shared between the 2G4 and 4F5 epitopes (Table 2, Fig. 4). Two of these residues Glu604 and Asp608 produce some of the strongest interactions with both antibodies. Glu604 forms three salt bridges, one ion pair and one hydrogen bond with 2G4, while it produces one ion pair with 4F5. Asp608 is involved in one ion pair with 2G4 and it forms two salt bridges with 4F5. The epitopes for 2G4 and 4F5 are located on different but adjacent surfaces of TPO with different orientations on the TPO structure. The coordinate superimposition of the two complexes clearly shows that 2G4 and 4F5 bind to two different epitopes located exclusively on the POD with different orientations relative to each other and to TPO (Fig. 5). The three TPO residues interacting with both 2G4 and 4F5 contribute to a common rim for the two antibody binding surfaces (Fig. 4C and D). As shown in Fig. 4, several residues from 4F5 and 2G4 epitopes correspond to the residues assigned to immunodominant region B (IDR-B) indicating that 2G4 and 4F5 bind to IDR-B (Le *et al.* 2015, Williams *et al.* 2018). The residues assigned to IDR-A (Le *et al.* 2015, Williams *et al.* 2018) are located on the TPO structure in five separate clusters (1–5) at some distance from each other (Fig. 4C and D). IDR-A clusters 1 and 2 on TPO and clusters 3, 4 and 5 are separated by a protruding area on the TPO surface (Fig. 4).

The 2G4 and 4F5 epitopes and the residues assigned to the IDR-A and IDR-B are located on the opposite surface of the TPO structure to the surface containing the entrance to the channel that gives access to the TPO enzyme active site (Fig. 5).

## Discussion

### Human TPO structure

TPO is a haem containing peroxidase enzyme homologous to other members of the animal haem-dependent peroxidase family such as myeloperoxidase (MPO) and lactoperoxidase (LPO) (Fiedler *et al.* 2000, Singh *et al.* 2009). The hydrophilic extracellular domain of TPO in both of our solved TPO-antibody complex structures shows TPO present as a monomer. In contrast, human MPO (hMPO) has been shown to form a dimer with a disulphide bond linking adjacent Cys153 residues (Davey & Fenna 1996). While hTPO has an equivalent residue to hMPO at Cys296 no dimer formation was observed in our structure which is in agreement with the monomeric TPO present in the insect cell culture supernatants before purification. However, this does not exclude the possibility that Cys oxidation or other modifications have prevented Cys bond formation during expression. Furthermore, there is a possibility that a dimer could be formed between two full-length, membrane-bound hTPO molecules through an intermolecular disulphide bond. The structure of TPO in both of our complexes is that of a globular protein with three domains in which the POD and CCP domain are linked by a disulphide bond such that rearrangement of the relative positions of the domains cannot occur. This is in contrast to previous suggestions, based on a low-resolution cryo-EM structure (20 Å resolution) and molecular dynamics simulations, that the POD and CCP domains could move on antibody binding to bring the IDR-A regions closer together (Williams *et al.* 2020). Our cryo-EM structure shows that the EGF domain of TPO is not disulphide bonded to the POD or CCP domains and this allows some flexibility for the TPO structure.

The TPO POD is classed as an all-alpha protein consisting of a central core of five helices and a covalently attached haem group. The percentage of residues with secondary structure is very low with 38 and 40% of the residues in the POD of TPO-4F5 and TPO-2G4, respectively, within the secondary structure. hMPO is similar to TPO in this respect and has only 42% of residues present in the secondary structure. The structure of the TPO CCP domain, also known as the sushi domain (Ichinose *et al.* 1990) or short consensus repeat, consists of the two faces of the β-sandwich of a typical sushi domain. It contains four invariant cysteine residues which form two disulphide bonds (Cys742-Cys-782 and Cys768-Cys794, Supplementary Fig. 2), a highly conserved tryptophan (Trp787), and conserved glycine and proline residues Gly786 and Pro792 (Reid & Day 1989). The CCP domain is found in a range of proteins involved in protein–protein interactions at the cell surface (Kato & Enjyoji 1991) and is thought to ensure the appropriate spatial arrangement of functional domains and impart a degree of flexibility to the stems of receptors. The structure of the TPO EGF domain is that of a typical EGF-like domain which is expected to contain three disulphide bonds (Wouters *et al.* 2005). In our structure, only two disulphide bonds are visible between Cys800-Cys814 and Cys808-Cys823 (Supplementary Fig. 2); however, only one of the other two TPO cysteine residues Cys825 is visible in the structure while Cys838 is not; therefore, the third disulphide bond in the EGF domain is not visible. EGF-like domains have been shown to be present in a large number of membrane-bound, extracellular-facing eukaryotic proteins and they can act as a spacer between cell surface proteins and can mediate protein–protein interactions (Campbell & Bork 1993).

A very low-resolution (20 Å) cryo-EM structure of the extracellular region of TPO in complex with the TR1.9 antibody (Williams *et al.* 2020) only allowed the domain locations to be discerned. However, using molecular dynamics simulations, the authors proposed three different orientations for the TPO molecule; *cis*, with the active site of the POD facing towards the thyrocyte membrane, *trans*, with the active site facing away from the membrane and extended where the CCP and EGF domains extend out from the POD. In our high-resolution cryo-EM structures, the domain orientation is clear and although it most closely resembles the proposed *cis* model it is not the same. The TPO CCP domain of the *cis* model is rotated further away from the POD domain than in our TPO cryo-EM structures, where the domains are closer to each other.

### Structure of TPO enzyme active site

The residues in both complexes which interact with the TPO haem group are similar with 20 interacting residues in the TPO-2G4 complex and 19 in the TPO-4F5 complex. Residues His239 and Arg396 in the TPO active site have been shown to play an important role in the TPO enzymatic reaction (Taurog & Wall 1998). His239 serves as an acid-base catalyst transferring a proton from the iron-bound oxygen atom of H_2_O_2_ to the second oxygen atom, which then leaves as a water molecule (Poulos & Kraut 1980). Arg396 polarises the bond between the two oxygen atoms which promotes the heterolytic cleavage of the bond (Rodríguez-López *et al.* 2001).

A superimposition of the active site of hTPO with other haem containing peroxidases, hMPO and bovine LPO (bLPO) showed they have similar structural features (Supplementary Fig. 4A, B, C, D and E). The haem groups are held by two covalent bonds between the carboxylate groups of aspartic acid (Asp238 in hTPO, Asp94 in hMPO and bLPO) and glutamic acid (Glu399 in hTPO, Glu242 in hMPO and bLPO) and the interaction between the proximal histidine (His494 in hTPO, His336 in hMPO and bLPO) with the iron ion (Supplementary Fig. 4). Furthermore, the three arginine residues (Arg396, 491 and 582 in hTPO and Arg239, 333 and 424 in hMPO and bLPO) that form salt bridge interactions with the carboxylate groups of the haem are in similar positions (Supplementary Fig. 4C). The residues surrounding the active sites (Gln235, Asp238, Arg396 and Glu399 in hTPO and Gln91, Asp94, Arg396 and Glu242 in hMPO and bLPO) occupy similar positions in the superimposition with the distal histidine (His239 in hTPO and His95 in hMPO and bLPO) located in the centre of the active site above the iron ion (Supplementary Fig. 4B, D and E).

### TPO calcium binding site

The calcium binding site of hTPO is similar to the calcium binding site observed in the structure of hMPO (Fiedler *et al.* 2000). Furthermore, the calcium binding site has been shown to be structurally important and conserved in the peroxidase family (Furtmüller *et al.* 2006). Mutating Asn96 in hMPO (equivalent to Asp240 in hTPO) to alanine or the equivalent substitution in hLPO has been shown to reduce enzyme activity almost completely (Shin *et al.* 2001).

### Interaction of 2G4 Fab and 4F5 Fab with TPO in the complexes

The cryo-EM structures of the complexes show the interaction of TPO with 2G4 and 4F5 in detail and indicate clearly that they react with different, distinct epitopes on TPO (Tables 2 and 3, Fig. 5). The 2G4 hMAB was derived from a patient with Hashimoto’s thyroiditis (Horimoto *et al.* 1992) and is a hTPO autoantibody*.* In contrast, 4F5 is a mouse antibody which displays some characteristics of hTPO autoantibodies, in particular, high-affinity binding to TPO and the ability to inhibit TPOAb binding to TPO (Hendry *et al.* 2001*b*). Consequently, the binding arrangements of 2G4 Fab with TPO and 4F5 with TPO in the complexes can be considered to be representative of at least some TPO autoantibodies. The TPO-antibody topography of the antibody binding interfaces and the strong interactions between 2G4 Fab with TPO and 4F5 Fab with TPO seen in the structures are consistent with the high-binding affinity (4.2 × 10^8^ and 4.4 × 10^9^ L/mol, respectively; Horimoto *et al.* 1992, Hendry *et al.* 2001*b*) of these antibodies.

Previous extensive experimental studies using the mouse or human antibodies defined the contributions of TPO residues to the IDRs (Ruf *et al.* 1989, Chazenbalk *et al.* 1993, Czarnocka *et al.* 1997, Guo *et al.* 1999, Guo *et al.* 2001, Estienne *et al.* 2002, Bresson *et al.* 2003, 2005, Gora *et al.* 2004, Rebuffat *et al.* 2006 Godlewska *et al.* 2012, Le *et al.* 2015, Williams *et al.* 2018). However, the nomenclature of IDR-A and IDR-B using mouse and human antibodies was inverted causing some confusion (Ruf & Carayon 2006, McLachlan & Rapoport 2008, Williams *et al.* 2018). Here, we refer to the IDRs as they were first defined using mouse antibodies (Ruf *et al.* 1989). The residues found contributing to IDR-A included amino acids: 225, 353–363, 377–386, 646, 707 and 713–720 of the POD and 766–775 of the CCP while the residues found to be important for IDR-B were clustered together on the POD and included amino acids: 597–604, 611–618, 620, 627 and 630 (Ruf & Carayon 2006, McLachlan & Rapoport 2008, Le *et al.* 2015, Williams *et al.* 2018).

In our cryo-EM structures, 2G4 and 4F5 bind exclusively to epitopes on the TPO POD and share several residues with the IDR-B. TPO residues 597–599, 601–604 and 611–612 are common for both, the 4F5 epitope and the IDR-B. TPO residues 604, 616, 620 and 627 are common for both, the 2G4 epitope and the IDR-B (Table 2, Fig. 4 and 5). Consequently, the 2G4 and 4F5 epitopes on TPO correspond to IDR-B (Ruf & Carayon 2006, McLachlan & Rapoport 2008, Le *et al.* 2015, Williams *et al.* 2018). Tryptic epitope excision analysis has indicated that the major 4F5 epitope included TPO aa 585–616 and this is consistent with the interactions seen in the cryo-EM structure (Thomas 2019). The contribution of TPO Glu604 to strong interactions with both 2G4 and 4F5 is in agreement with experimental studies indicating that Glu604 is involved in producing a functional epitope within IDR-B (Dubska *et al.* 2006). However, Glu604, Asp624 or Lys627 which are included in the 4F5 and/or 2G4 epitopes were found not to be important for the binding of three different mMABs produced by immunisation with purified hTPO (Godlewska *et al.* 2012). These apparent discrepancies between the experimental studies and the interactions seen in the complexes of TPO with 2G4 and 4F5 may be related to the conformational nature of TPO epitopes where although the antibodies bind to a similar region on TPO the actual contact residues for different antibodies may vary (Baker *et al.* 1994, Czarnocka *et al.* 1997, Grennan Jones *et al.* 1999, Guo *et al.* 1999, Hobby *et al.* 2000, Bresson *et al.* 2005). This is a similar scenario to the characteristics of epitopes for other autoantibodies which may recognise a common region on the antigen molecule and yet make contacts with different residues in that region (Grennan Jones *et al.* 1999, Hayakawa *et al.* 2002, McLaughlin *et al.* 2015, Furmaniak *et al.* 2015).

The residues assigned to IDR-A are located in five separate clusters (1–5) on our cryo-EM TPO structure (Fig. 4). The distances between the different clusters 1–5 are too big to be contacted at the same time by an antibody Fab fragment or by an intact antibody. The IDR-A clusters 1 and 2 and clusters 3, 4 and 5 are separated by a protruding area on the TPO surface (Fig. 4), and the two closest atoms from clusters 3 and 5 are more than 30 Å apart. However, the longest distance within the variable combining region of 2G4 is 28 Å. Due to this topography, the different IDR-A clusters on the TPO structure could not be contacted by one antibody. Consequently, the IDR-A clusters of residues may be involved in forming several heterogeneous epitopes for different TPOAbs while the IDR-B is a more compact antibody binding region on TPO.

In the cryo-EM structures, 2G4 and 4F5 do not form interactions with the CCP or EGF domains (Fig. 4 and 5). Previously, molecular dynamic simulations suggested that conformational TPOAb epitopes may be formed by merging the epitopes on the POD with those on the CCP or EGF domains through intra-molecular movements of the domains adopting different conformations upon antibody binding (Williams *et al.* 2018, 2020). However, this scenario is unlikely as any movement of the TPO domains relative to each other would be prevented by a disulphide bond between POD Cys146 and CCP Cys756 which is clearly visible in the cryo-EM structures (Fig. 2B).

Both 2G4 and 4F5 bind on the opposite side of the TPO molecule to the entrance of the enzyme active site and the calcium binding site and this corresponds with the antibodies inability to inhibit TPO enzyme activity (Gut *et al.* 2000). The activity of anti-thyroid drugs, which bind to the TPO enzyme active site, would also not be expected to be affected by binding of TPOAb.

Our cryo-EM structures of TPO-antibody complexes provide key insights into the interactions between TPO and TPOAbs at the molecular level. As such, they further our understanding of the molecular basis of the autoimmune response to TPO and should help in developing new strategies to prevent AITD. In addition, the solved structures can guide the development of new inhibitors of TPO enzyme activity for therapeutic applications.

## Supplementary Material

Supplementary Material

## Declaration of interest

RSR Ltd is a developer and manufacturer of *in vitro* medical diagnostics. The work was carried out while all authors were employees of RSR Ltd.

## Funding

The work was funded by RSR Ltd.
