# Supplementary material for: Cryo-electron microscopy structures of human thyroid peroxidase (TPO) in complex with TPO antibodies
Source: J Mol Endocrinol. 2023 Jan 24;70(3):e220149. doi: 10.1530/JME-22-0149 (PMC9986399; doi:10.1530/JME-22-0149)

Supplementary Table 1 Cryo-electron microscopy data collection, refinement and validation statistics

	TPO-4F5	TPO-2G4
**Data collection and processing**		
Detector	Falcon 3	Falcon 3
Magnification	96k	96k
Voltage (kV)	300	300
Flux on detector (e/pix/sec)	0.67	0.67
Electron exposure on sample (e–/Å^2)	45.41	43.86
Target defocus range (μm)	0.9-2.4	0.9-2.4
Calibrated pixel size (Å)	0.827	0.827
Symmetry imposed	C1	C1
Extraction box size (pixels)	300	340
Initial particle images (no.)	313781	191172
Final particle images (no.)	66270	37338
**Refinement**		
Map resolution at FSC=0.143 (Å)	3.40	3.92
Model composition		
Non-hydrogen atoms	8972	8942
Protein residues	1126	1137
Nucleotides	-	-
B factor (Å^2)		
Protein	125.47	129.81
Ligand	-	-

	TPO-4F5	TPO-2G4
R.m.s deviations		
Bond lengths (Å)	0.020	0.021
Bond angles (°)	2.444	2.510
Validation		
Molprobity score	3.75	3.83
Clashscore	104.96	114.61
Poor rotamers (%)	8.69	11.88
Ramachandran plot		
Favored (%)	89.79	91.73
Allowed (%)	97.42	97.98
Disallowed (%)	2.58	2.02

The model was validated using PROCHECK, Verify3D and MolProbity (Supplementary Table 1). PROCHECK reported 47 bad contacts and an overall G-factor of -0.27 for the TPO-2G4 complex while there were 60 bad contacts and an overall G-factor of -0.21 for the TPO-4F5 complex. Verify3D reported that 89.00% and 93.09% of the residues in the TPO-2G4 complex and TPO-4F5 complex respectively had an average 3D-1D score>=0.2. MolProbity reported a Rama distribution Z-score of -1.11 and -1.78 for the TPO-2G4 complex and TPO-4F5 complex respectively. The resolution of the cryo-EM density map corresponding to the CCP and EGF domains is poorer (6-7 Å) compared to the rest of the complex structures (Supplementary Figure 1G and 1I) and these two domains account for most of the clashes and outliers present in the final model.

Supplementary Table 2 TPO - antibody complex interface areas.

	molecule/chain	2G4	4F5
**TPO - Fab interface**	TPO	1070 Å^2	1007 Å^2
	Fab	1078 Å^2	952 Å^2
**TPO - light chain interface**	TPO	502 Å^2	405 Å^2
	Light chain	521 Å^2	423 Å^2
**TPO - heavy chain interface**	TPO	639 Å^2	678 Å^2
	Heavy chain	699 Å^2	663 Å^2

Supplementary Figure 1

Cryo-electron microscopy (cryo-EM) images and electron density maps of the TPO-2G4 and TPO-4F5 complexes.

A Cryo-EM imaging on Titan Krios showing particles of the TPO-4F5 complex. 1,726 movies were collected and WARP identified 313,000 particles.

B Cryo-EM imaging on Titan Krios showing particles of the TPO-2G4 complex. 1,949 movies were collected and WARP identified 191,000 particles.

C Data analysis in CryoSPARC showing 2D classification. Average images of the 2D projections of the particles of TPO-4F5 complex picked by WARP.

D Data analysis in CryoSPARC showing 2D classification. Average images of the 2D projections of the particles of TPO-2G4 complex picked by WARP

E The final TPO-2G4 map is shown in 2 views rotated by 180°. The complex structure was solved at a global resolution of 3.92Å.

F The final TPO-4F5 map is shown in 2 views rotated by 180°. The complex structure was solved at a global resolution of 3.40Å.

G Cryo-EM map of the TPO-2G4 complex. The regions of the cryo-EM density map are coloured by local resolutions from 3.35Å (red) to 8.6Å (blue). The resolution of the overall density map is 3.92Å resolution.

H Fourier shell correlation (FSC) curve calculated from two independent reconstructions of the TPO-2G4 complex map by CryoSPARC.

I Cryo-EM map of the TPO-4F5 complex. The regions of the cryo-EM density map are coloured by local resolutions from 2.88Å (red) to 7.0Å (blue). The resolution of the overall density map is 3.40Å resolution.

J Fourier shell correlation (FSC) curve calculated from two independent reconstructions of the TPO-4F5 complex map by CryoSPARC.

Supplementary Figure 1 A

TPO-4F5 Complex

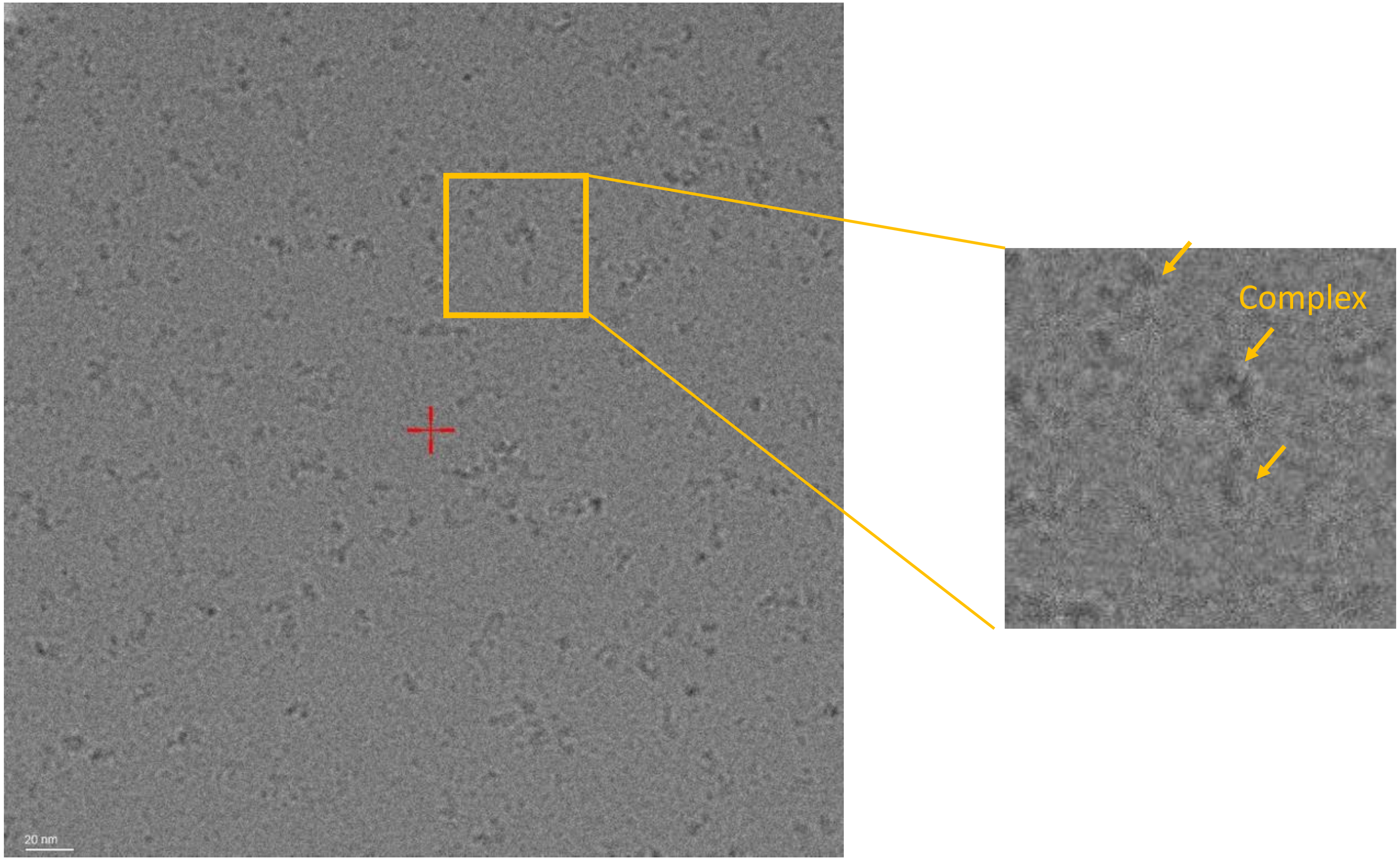

Supplementary Figure 1 B

TPO-2G4 complex

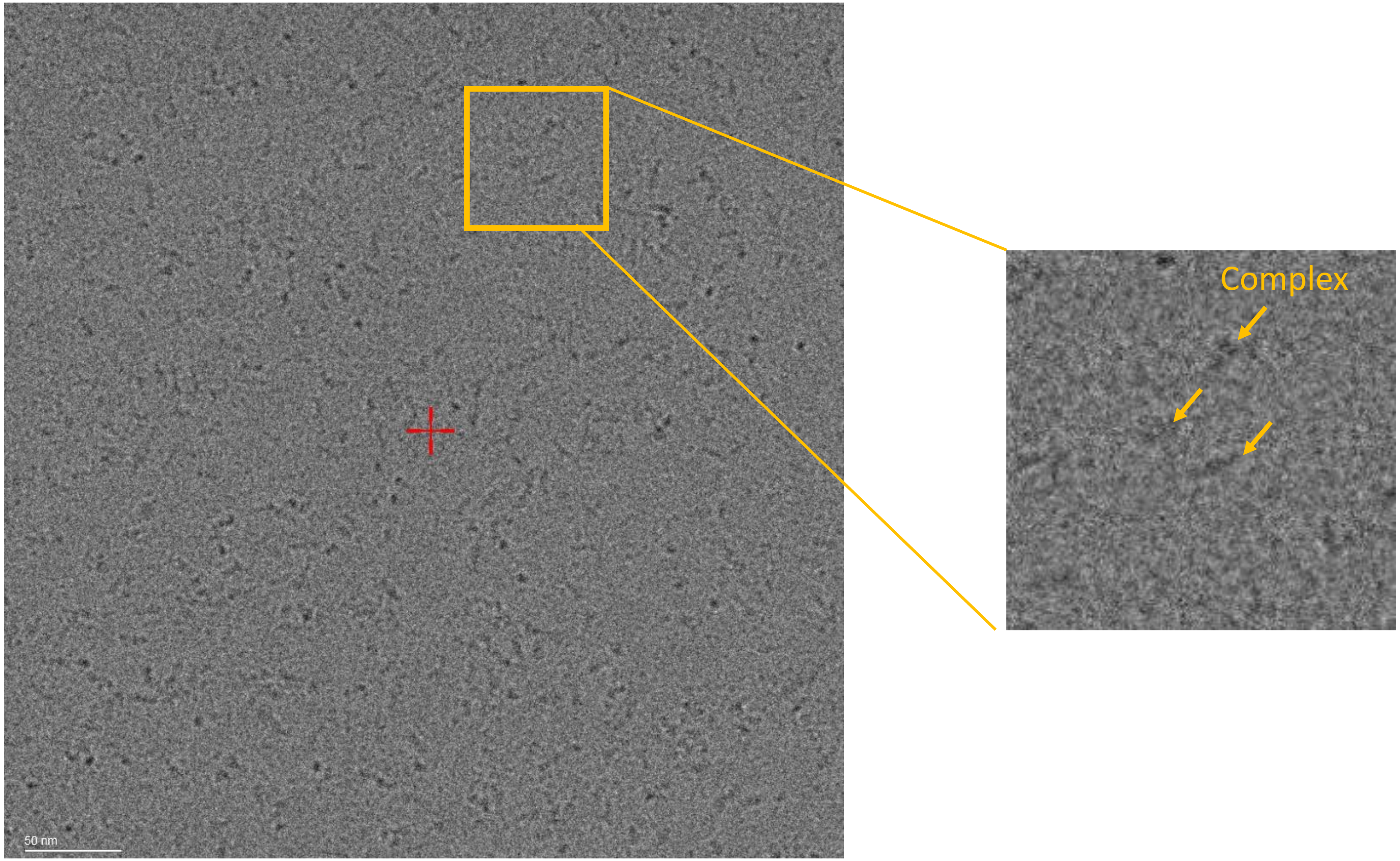

Supplementary Figure 1C

TPO-4F5 complex

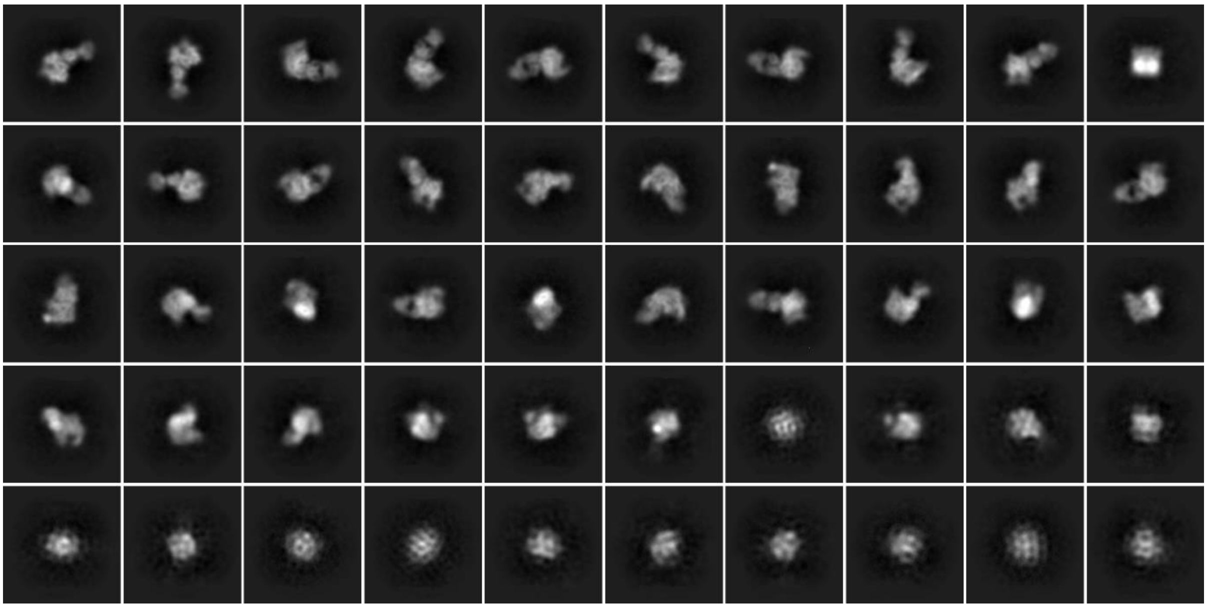

Supplementary Figure 1D

TPO-2G4 complex

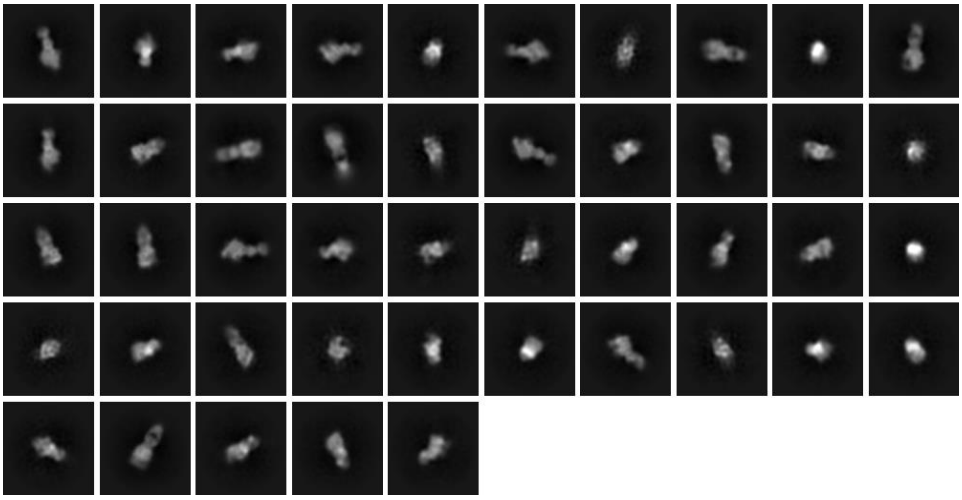

Supplementary Figure 1

E

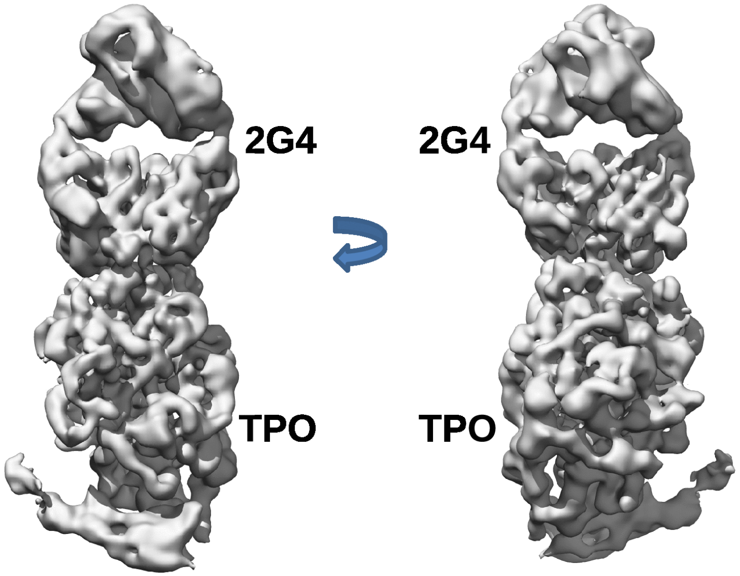

F

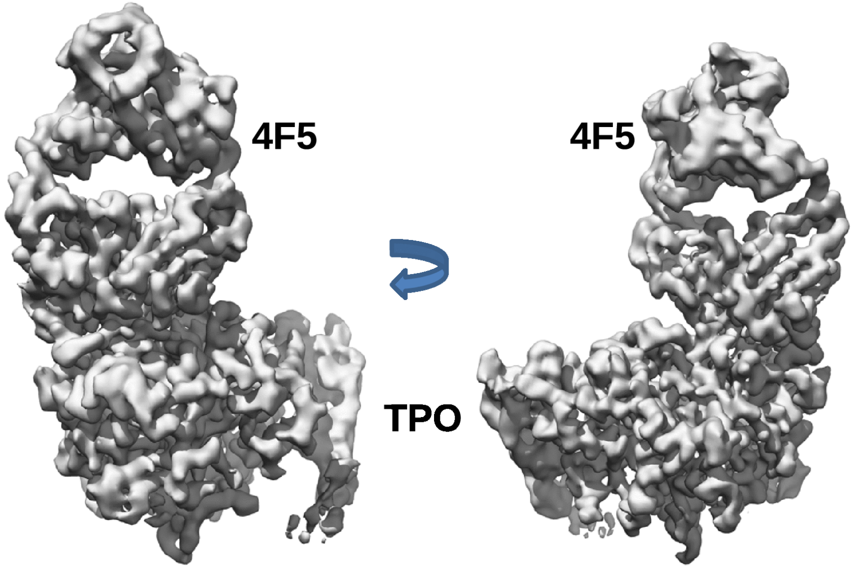

Supplementary Figure 1

G

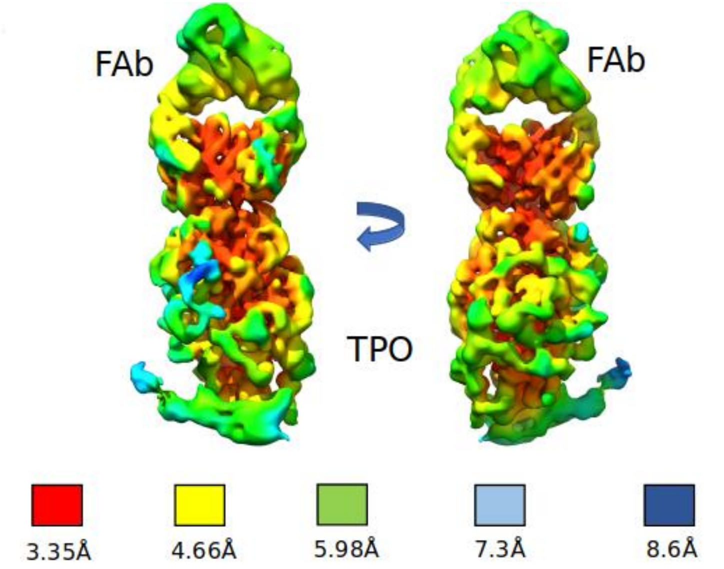

H

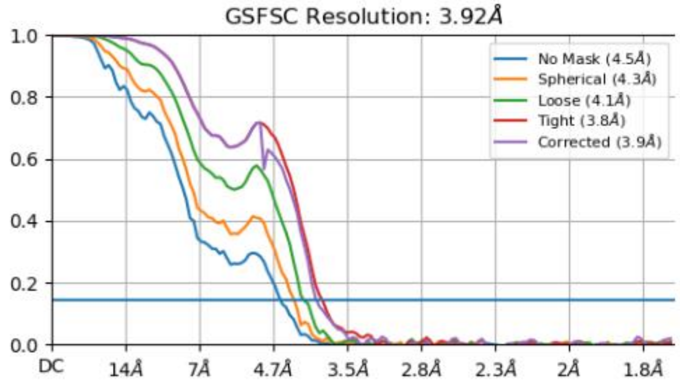

I

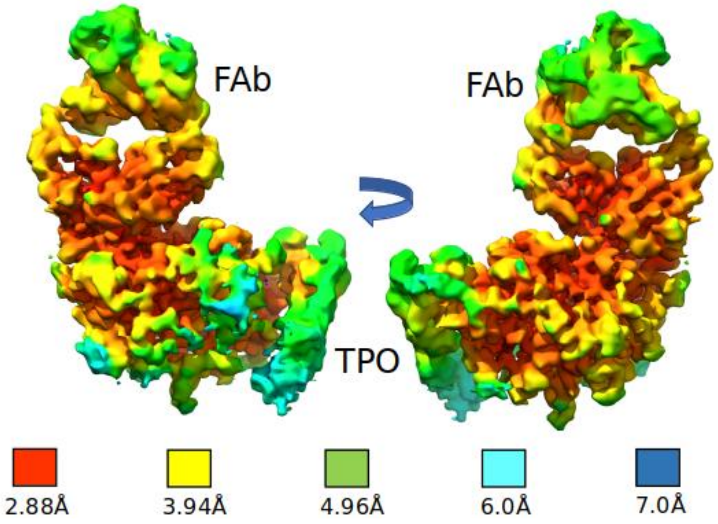

J

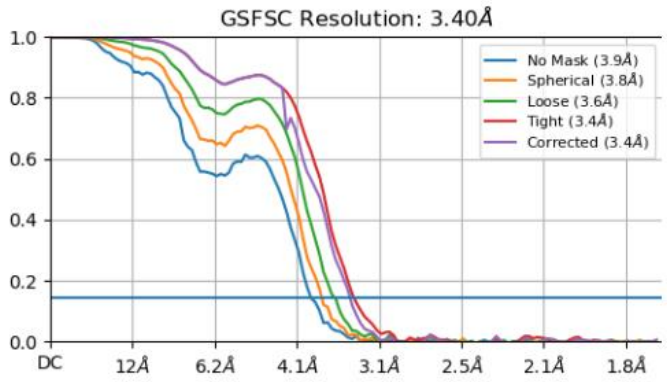

Supplementary Figure 2

TPO structure (amino acids Leu119 to Asp827). The structure is in a cartoon representation with the disulphide bonded cysteine residues and glycosylation sites in ball and stick representation with oxygen in red, nitrogen in blue and sulphur in yellow. The glycans are shown in ball and stick representation in purple. The N- and C-termini, POD, CCP and EGF domains, the haem, the calcium ion and the disulphide bond between Cys146 in the POD and Cys756 in the CCP domain are marked. The CCP and EGF domains have been enlarged to show the disulphide bonding arrangements.

Supplementary Figure 2

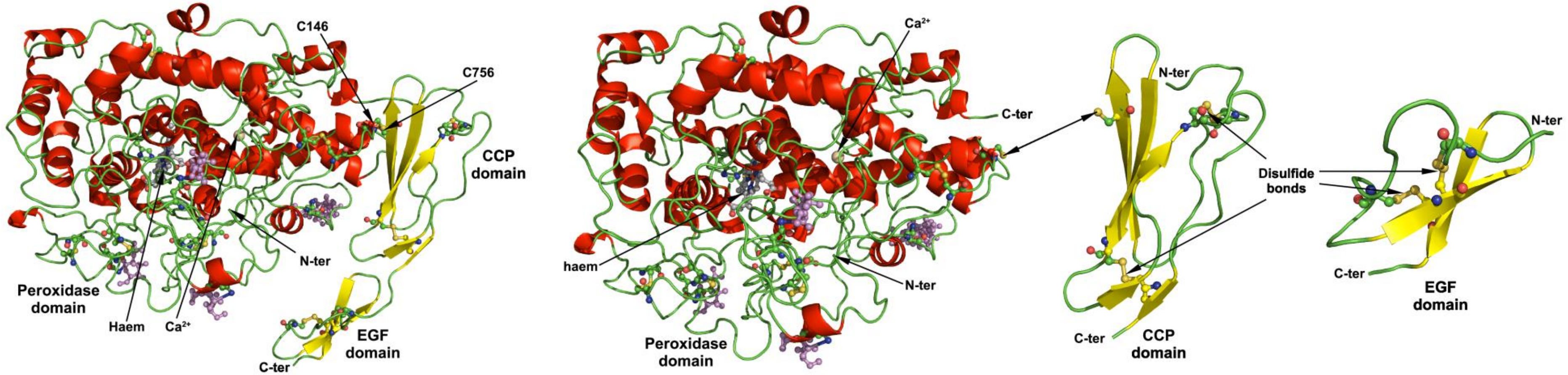

Supplementary Figure 3

The structure of mouse monoclonal antibody bound to TPO showing the calcium binding site. The structure is in a cartoon representation with the calcium binding site enlarged showing the calcium ion binding to TPO residues Asp240, Thr321, Phe323, Asp325 and Ser 327 in ball and stick representation with oxygen in red and nitrogen in blue. The calcium ion binds to TPO, away from the 4F5 binding site, with pentagonal bipyramidal geometry. The disulphide bonded cysteine residues, sulphur in yellow, and glycosylation sites are in ball and stick representation with the glycans in purple.

Supplementary Figure 3

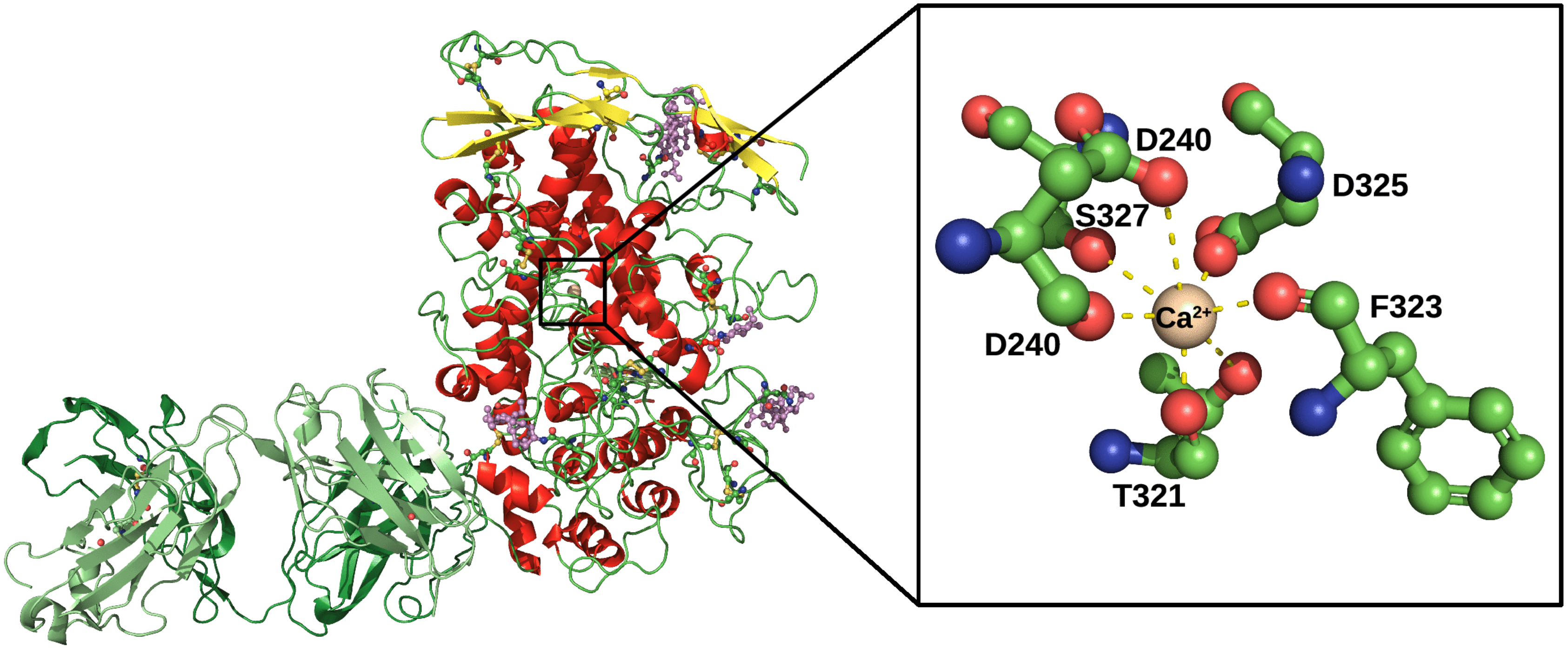

Supplementary Figure 4

Superimposition of the structures of human thyroid peroxidase (hTPO), human myeloperoxidase (hMPO) and bovine lactoperoxidase (bLPO).

A The active sites of hTPO (green), hMPO (light blue) and bLPO (dark blue) structures are shown with the haem binding site and the calcium ion marked.

B Residues that are covalently linked to the haem group are shown with the proximal (His494) and distal (His239) histidine residues marked.

C Arginine residues that make salt bridge interactions with the carboxylate groups of the haem are shown.

D and **E** Residues lining the active site are shown in two different views rotated by approximately 90º along a horizontal axis.

The haem and functionally important residues are shown in a "sticks" representation with oxygen atoms in red, nitrogen atoms in blue and carbon atoms in the same colour as the peroxidase to which they belong as above. Residue numbers correspond to hTPO.

Supplementary Figure 4

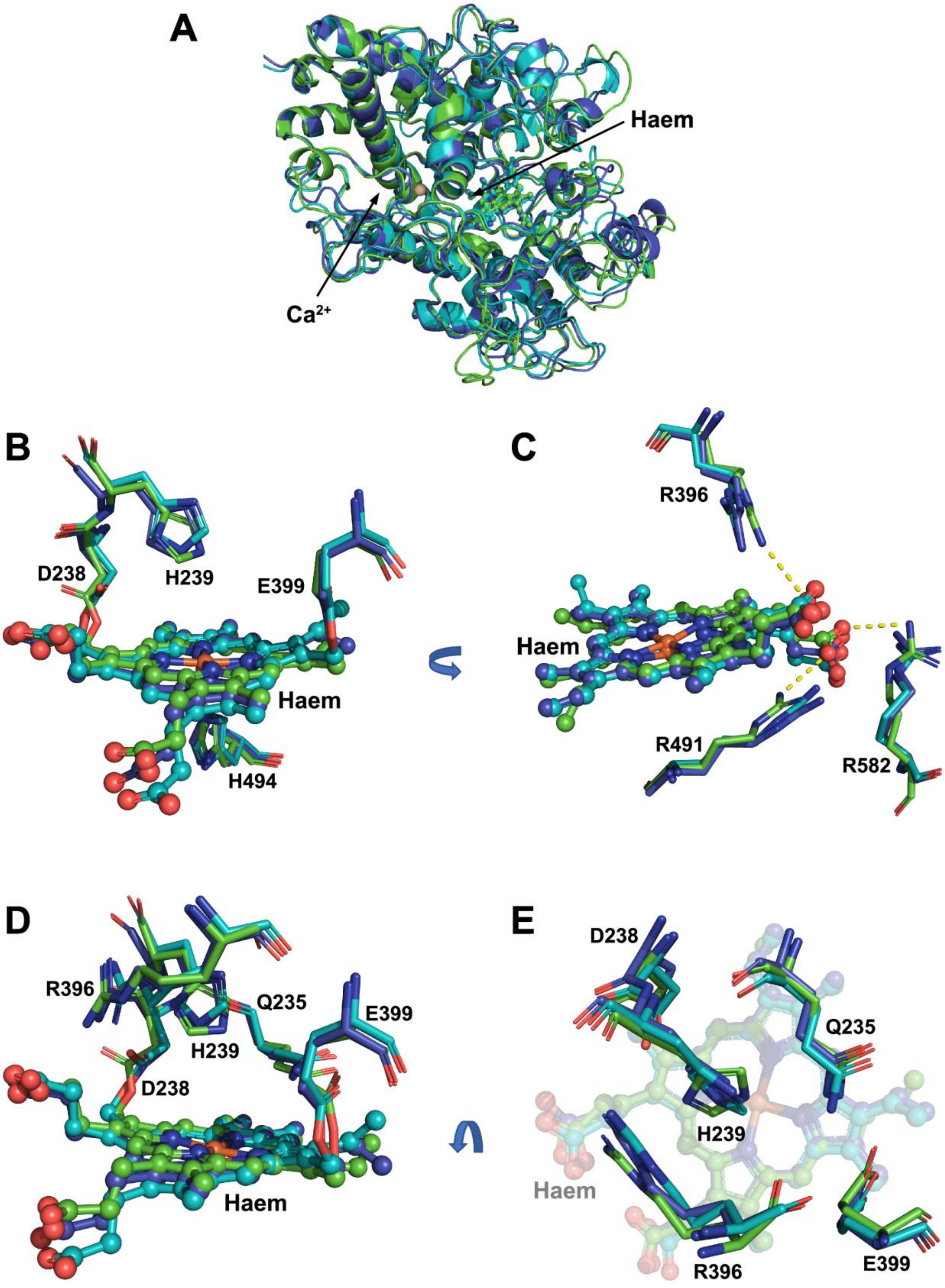

Supplement: Supplementary Material [file supplementary_material.pdf]
